# Membranes Based on PTMSP/PVTMS Blends for Membrane Contactor Applications

**DOI:** 10.3390/membranes12111160

**Published:** 2022-11-17

**Authors:** Denis Kalmykov, Alexey Balynin, Alexey Yushkin, Evgenia Grushevenko, Stepan Sokolov, Alexander Malakhov, Alexey Volkov, Stepan Bazhenov

**Affiliations:** A.V. Topchiev Institute of Petrochemical Synthesis RAS, 29 Leninsky Prospekt, 119991 Moscow, Russia

**Keywords:** carbon dioxide, membrane contactor, PTMSP, PVTMS, oxygen removal

## Abstract

In this work, perspective polymeric materials were developed for membrane contactor applications, e.g., for the dissolved oxygen removal from amine CO_2_ capture solvents. Several polymeric blends based on poly[1-trimethylsilyl-1-propyne] (PTMSP) and poly[vinyltrimethylsilane] (PVTMS) were studied. The gas and water vapor sorption and permeability coefficients for the PTMSP/PVTMS blend membranes at different PVTMS contents (0–100%) were obtained under temperatures of 30 and 60 °C for the first time. As the PVTMS content increases, the O_2_ and CO_2_ permeabilities decrease by 160 and 195 times at 30 °C, respectively. The fractional accessible volume of the polymer blends decreases accordingly. The transport of the CO_2_ capture solvent vapors through the PTMSP/PVTMS blend membranes were determined in thermo-pervaporation (TPV) mode using aqueous monoethanolamine (30%), N-methyldiethanolamine (40%), and 2-amino-2-methyl-1-propanol (30%) solutions as model amine solvents at 60 °C. The membranes demonstrated high pervaporation separation factors with respect to water, resulting in low amine losses. A joint analysis of the gas permeabilities and aqueous alkanolamine TPV data allowed us to conclude that the polymer blend composition of PTMSP/PVTMS 70/30 provides an optimal combination of a sufficiently high oxygen permeability and the pervaporation separation factor at a temperature of 60 °C.

## 1. Introduction

The problem of the influence of greenhouse gases, in particular, carbon dioxide, on global climate change has remained relevant for decades of modern history [1]. Currently, programs to reduce CO_2_ emissions are becoming social, economic, and political trends [2,3]. Currently, CO_2_ capture and storage (CCS) is considered to be the most promising strategy for reducing carbon dioxide emissions. Among the industrial CO_2_ capture methods, the most mature is the absorption process using aqueous alkanolamine solvents. An important step of this process is the CO_2_ stripping from the solvent, which is carried out at elevated temperatures (above 100–120 °C), which leads to solvent degradation, significantly accelerated in the presence of dissolved oxygen [4]. 

Table 1 shows the typical flue gas composition and the corresponding kinetic diameters of the gas molecules. In this case, the O_2_ content in the flue gas is no more than 4%. At the same time, off-gases in metallurgy, petrochemistry, and other industries can contain up to 15% of O_2_ [5]. Another source of O_2_ which can dissolve in amine solvent during the CO_2_ capture process may be the leaks in the amine system or its non-tight storage (in contact with atmospheric air) [6]. 

Such attention to the O_2_ content in the amine solvents is associated with its negative impact on the CO_2_ capture process. Firstly, the increased O_2_ content significantly intensifies the corrosion activity in amine media [8,9,10,11,12,13]. Secondly, O_2_ contributes to the oxidative degradation of amine solvents, which leads to their significant losses (from 0.2 to 3.65 kg per ton of CO_2_ which is captured [14]). The oxidation process involves many reactions of free oxygen radicals with amine molecules or amine carbamates [5,15,16]. In this case, a wide range of degradation products is formed, such as carboxylic acids, amino acids, amides, amines, aldehydes, ammonia, etc. [15]. This fact is confirmed by the field tests of various amine solvents on 18 pilot post-combustion CO_2_ capture plants [5]. Oxidation products lead to declines in the solvent physicochemical properties, its foaming, equipment erosion, and contamination. Some of the products irreversibly bind the active amine into unregenerated compounds (in particular, heat-stable salts or HSS), which accumulate in the system and reduce the overall productivity of the amine system [4,17,18]. Being strongly corrosive [4,16], HSS increases the content of the corrosion products in a solvent (iron, chromium, nickel ions, etc.), which, in turn, catalyze a further oxidation of amines [17,18]. It leads to an autocatalytic reaction of an oxidative degradation of the solvent. 

Membrane technologies are promising alternatives for separation tasks in the field of CCS [19], and, in particular, for the removal of O_2_ from an aqueous media [20], due to a high energy efficiency, compactness, and modularity. For example, Liqui-Cel^®^ (3M) membrane contactors based on hydrophobic porous polypropylene hollow fiber membranes are successfully used to remove O_2_ and other gases from water [21,22,23,24,25]. A membrane contactor is an apparatus for carrying out separation processes in which the membrane acts as the interface between the two phases, excluding their mutual dispersion. Porous membranes are actively investigated for membrane contactor applications. However, non-porous membranes with thin dense layers based on highly permeable rubber [26] or glassy [27,28] polymers are more suitable in view of preventing a membrane wetting effect. 

The glassy polymer poly[1-trimethylsilyl-1-propine] (PTMSP) has record-high gas permeability coefficients. PTMSP is shown to be promising as a selective layer material for composite membrane contactors designed for a CO_2_ capture [28,29,30]. The O_2_ permeability coefficient for PTMSP is up to the 14,800 barrer [31]), thus this polymer is also suitable as a selective layer material for an amine solvent deoxygenation in composite membrane contactors. However, it is obvious that PTMSP also has high CO_2_ and water vapor permeabilities. Thus, during the deoxygenation of aqueous amine solutions, the competitive transport of O_2_, CO_2_, and solvent vapors is inevitable. The modification of PTMSP to “tune” its transport properties is therefore needed.

Recent studies on the membranes from the polymer blends have demonstrated the potential of a blending approach for a “fine-tuning” of the fractional free volume (FFV) of the polymeric membrane materials, resulting in a tailoring of their transport properties [32]. In particular, the work [33] analyzed the gas permeabilities of a wide range of polymer blends from polyimide, polybenzimidazole (PBI), poly(vinyl alcohol) (PVA), and polymers of intrinsic microporosity (PIMs). The work [34] refers to the permeabilities of blends of polyamide, polyethylene glycol (PEG), polyurethane, and others. The gas transport properties of the polymer blends are also studied in the papers [35,36]. It is shown that the addition of highly permeable glassy polymer poly[vinyltrimethylsilane] (PVTMS) to PTMSP can stabilize it transport properties in a gas separation [37] and the membrane contactors for the CO_2_ desorption from an amine solvent [38]. However, all these papers do not include a comprehensive study of the polymer blends toward their applicability as a selective layer for a composite membrane gas–liquid contactor designed for the deoxygenation of an amine solvent. In this work, a complete and in-depth study of the PTMSP/PVTMS blend membranes is presented for the first time with a focus on their morphological, gas and vapors sorption, and transport properties, as well as the amine solvent vapor permeation data.

## 2. Materials and Methods

### 2.1. Materials and Reagents

A commercial polymer PTMSP (SSP-070, lot 9D-35578, MW 250 × 103 g/mol, Gelest, Inc. (Morrisville, PA, USA)) and a commercial PVTMS sample with a density of 0.86 g/cm^3^ were used. Monoethanolamine (MEA, LLC TD HIMMED (Moscow, Russia)), N-methyldiethanolamine (MDEA, manufacturer of GC Sintez OKA (Nizhny Novgorod region, urban district city of Dzerzhinsk, city of Dzerzhinsk, Russia)), and 2-Amino-2-methylpropane-1-ol (AMP, manufacturer Sigma-Aldrich (Saint Louis, MI, USA)) were used for the preparation of the model amine solvents. Carbon dioxide, nitrogen, and oxygen (MGPZ, Moscow, Russia) were used for the permeability and sorption measurements.

**Figure 1:**
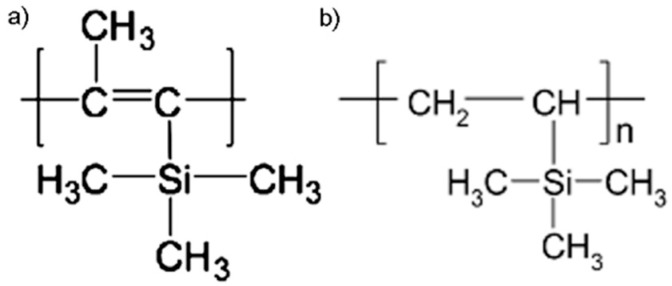
Structural formulas of the polymers PTMSP (**a**) and PVTMS (**b**) used in this work.

Figure 1 shows the structural formulas of the PTMSP and PVTMS polymers used in the work. Both polymers are similar in chemical nature: both polymers have a hydrocarbon main chain and a trimethylsilyl side substituent. At the same time, PTMSP is a record-breaking highly permeable polymer. The gas permeability of PVTMS is more than two orders of magnitude lower, whereas the selectivity of the gas separation of the gases is higher for PVTMS than for PTMSP [39].

The properties of polymer composite materials PTMSP/PVTMS were studied using homogeneous dense membranes, which are films with a thickness of 25–45 microns. A number of PTMSP/PVTMS solutions were prepared (see Table 2) by mixing the solutions of pure polymers in chloroform with a concentration of 1 wt%. The solutions were filtered under a pressure of nitrogen using double filter paper with a continuous concentration control by measuring the solid residual. Membranes from the PTMSP/PVTMS blend were obtained by pouring the corresponding solutions on commercial cellophane in a metal frame. The cast film was covered with a Petri dish to ensure a slow evaporation of the chloroform for 7 days at room temperature, followed by drying in the oven at 40 °C until reaching a constant sample weight. The films were treated with n-butanol, ethanol, and water–ethanol mixtures in accordance with the standard protocol [40]. All the polymer films were visually transparent. The thickness of the films was determined by the Mitutoyo^®^ 273 Quick Step (Neuss, Germany) electron micrometer with an accuracy of ±2 microns.

### 2.2. Measurements of Fractional Accessible Volume

The value of the fractional accessible volume (FAV) of the composite polymer materials was determined by the extended hydrostatic weighing method according to the methodology presented in [41]. The method is based on comparing the weight of a polymer film sample in non-wetting and wetting liquids. Distilled water and ethanol, respectively, were used. Membrane samples were used in the form of disks. A loop of a fishing line was attached to the edge of the disc to fix the load. The weight of the initial sample without a fishing line and with it was measured using Sartorius Analytical scales (Goettingen, Germany) with an accuracy of ±0.2 mg. The weight of the fishing line did not exceed 2 mg. After measuring the weight in the air, the film was placed in a closed container with distilled water for 24 h. After soaking, the weight of the sample in water was measured. To do this, a metal weight was attached to the sample, the weight of which in water was also previously measured. After measuring the weight of the sample with the load, the weight of the sample was determined by the difference between the measured value and the weight of the load. After that, the test sample was placed for 24 h in a container with ethanol, and the procedure for measuring the characteristics of the sample was repeated. The liquid density was monitored using a hydrometer (±0.001 g/cm^3^).

### 2.3. Gas Permeability Measurements

The transport properties of the polymer blend membranes were determined by measuring the gas permeability coefficients (P) of the films for pure gases: O_2_, CO_2,_ and N_2_. A manometric method for determining *P* using an automatic high-precision setup (HZG Gas and Vapor Permeability Test Unit, Geesthacht, Germany) was used. The effective area of the samples was 13.8 cm^2^. The experiments were carried out at temperatures of 30.0 ± 0.1 °C and 60.0 ± 0.1 °C (the maximum temperature of a carbonized amine solvent at the outlet of the absorption column in the CO_2_ capture system) at a gas pressure supplied to the membrane of 0.2–0.8 bar. Before measuring, the films were kept at these temperatures for at least 2 h to stabilize the transport properties. The gas permeability coefficient (*P*_i_) was expressed in barrers (1 barrer = 10^−10^ Ncm^3^·cm/(cm^2^·s·cm Hg.) and was determined by a linear extrapolation of the experimental data to zero the transmembrane pressure value. The ideal selectivity of the films was determined by the ratio of the permeability coefficients of individual gases (α = *P*_1_/*P*_2_).

### 2.4. Gas and Water Vapor Sorption Measurements

The sorption isotherms of O_2_, CO_2,_ and H_2_O were measured using the XEMIS-002 gravimetric sorption analyzer (Hiden Isochema, Warrington, UK) at temperatures of 30 and 60 °C and pressures up to p = 1 atm (for gases) or up to p/p_0_ > 0.75 (for water).

Before starting the measurement, the film samples were vacuumed at 60 °C for 24 h to remove the solvent residues. Further, by the method of a helium calibration, described in detail in the work of [42], the skeletal (pycnometric) densities of the samples necessary to account for the buoyant force acting on them were determined. The values of the gas and water vapor densities used in the work at different pressures and temperatures were taken from the NIST REFPROP V database.9.1 (accessed on 20 August 2022). At the pressures used in the experiment (less than 1 atm), the gas density is negligible compared to the density of the sorbed phase [42], which makes it possible to equate the values of excess and absolute absorption.

Each sorption isotherm was obtained at least 3 times to control the convergence of the results. Between measurements, the sample was vacuumed for 2 h at the temperature of the experiment. According to the sorption kinetics, the time for the mass of the sample to reach a stationary value did not exceed 20 min.

### 2.5. Differential Scanning Calorimetry

Calorimetric studies were performed in argon with calorimeter Mettler DSC823 (Mettler-Toledo GmbH, Columbus, OH, USA) at a scanning rate of 10 deg∙min−1 in the range of 140–100 °C. The glass transition can be observed as the midpoint of a step in the baseline of the measurement DSC curve.

### 2.6. Scanning Electron Microscopy (SEM)

Scanning electron microscopy (SEM) was used to characterize the structure and morphology of the polymer blend membranes. SEM was carried out on a Thermo Fisher Phenom XL G2 Desktop SEM (Waltham, MA USA). Cross-sections of the membranes were obtained in liquid nitrogen after a preliminary impregnation of the specimens in isopropanol. A thin (5–10 nm) gold layer was deposited on the prepared samples in a vacuum chamber (≈0.01 mbar) using a desktop magnetron sputter, the Cressington 108 Auto Sputter Coater (Watford, UK). The accelerating voltage during the image acquisition was 15 kV.

### 2.7. CO_2_ Solvent Vapor Transport with Thermo-Pervaporation of Aqueous Alkanolamine Solutions

**Figure 2:**
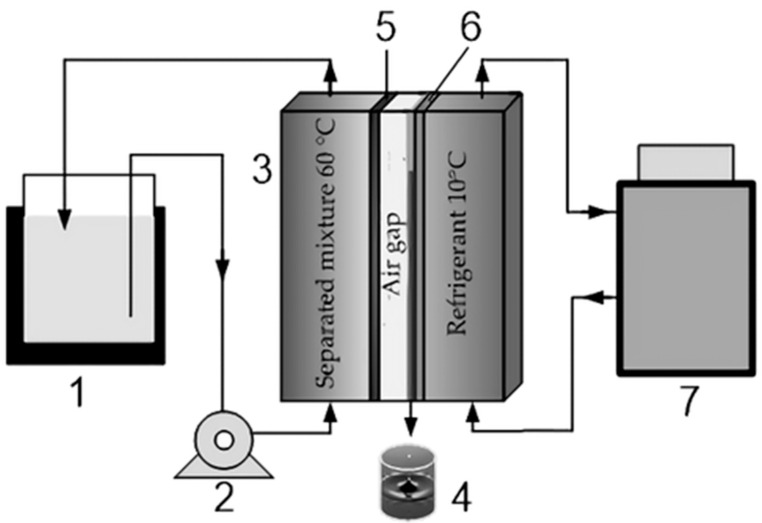
The scheme of a TPV unit: 1—a thermostat with a separable mixture; 2—a peristaltic pump; 3—a membrane module; 4—a permeate collector; 5—a membrane, 6—a metal condensation surface; and 7—cryothermostat.

During the O_2_ removal process from the amine solvents in the gas–liquid membrane contactor, the competitive transport of solvent vapors along with the targeted transport of O_2_ molecules is inevitable. In this regard, the transport properties of the polymer blend membranes toward solvent vapors were studied. For this purpose, the thermo-pervaporation (TPV) of pure aqueous amine solutions (MEA 30 wt%, MDEA 40 wt%, and AMP 30 wt%) was carried out. All the solutions were prepared from pure alkanolamines and distilled water by the weight method. The experiments to study the properties of the polymer blend membranes were carried out on the lab-scale unit shown in Figure 2. Two liquid circuits of different temperatures were connected to the TPV module (3). The first circuit with refrigerant is closed to a cryostat (7). The second circuit consists of a peristaltic pump (2) and a container with an amine solution (2 l flask) located in a thermostat (1) which heats the solution to a predetermined temperature. The TPV module consists of two mirror-symmetrical parts separated by a membrane (5) and a condensation surface (6). There is an air gap between (5) and (6). The permeate was condensed on a metal plate (6) and removed from the TPV module by gravity into a permeate collector (4). The effective area of the membrane was 25.5 cm^2^. The TPV process was carried out at T = 60 (±0.2) °C, while the pressure in the submembrane space was equal to the atmosphere. The permeate was condensed at T_cond_ = 10 °C. The width of the air gap was 2.5 mm. The total permeate flow J, kg/m^2^·h, was determined by the method according to the Formula (1).

(1) $$J=\frac{m}{S\cdot t},$$

where m is the total mass of the permeate (kg) penetrated through a membrane with an area of S (m^2^) during t, h. The separation factor was determined by the Formula (2).

(2) $$\alpha =\frac{y_{o}\cdot x_{w}}{y_{w}\cdot x_{o}},$$

where *x_o_* and *x_w_* are the mass fractions of alkanolamine and water in the separated mixture, and *y_o_* and *y_w_* are the mass fractions of alkanolamine and water in permeate.

Permeate and retentate were analyzed by ion exchange chromatography using the “Aquilon-Stayer-Ì” (Aquilon, Podolsk, Russia) system (chromatographic cation exchange column Shodex ICSI-50 4E (Showa Denko KK, Tokyo, Japan) eluent, an aqueous solution of nitric acid with a concentration of 0.004 mol/dm^3^), equipped with an EMCES 21 electric suppressor and a conductometric detector CD-510 with an electrical conductivity measurement accuracy of 0.1%.

## 3. Results and Discussion

### 3.1. Polymer Blend Materials Research

**Figure 3:**
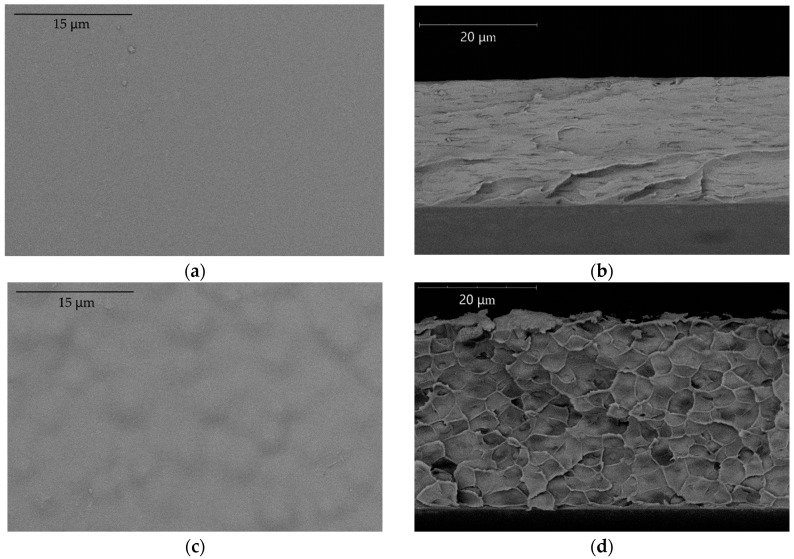
Micrograph of the surface (**a**) and cross-section (**b**) of the blend #4 and the surface (**c**) and cross-section (**d**) of the blend #5.

The properties of pure PTMSP and PVTMS materials and their blends were studied using SEM and DSC. It was revealed that with a complete uniformity of pure PTMSP and PVTMS films, their blends exhibit a more complex delaminating structure, especially at a higher PVTMS content. At the same time, it is clearly noticeable that with an increase in the PVTMS content, the nature of the resulting structures changes. Most likely, this change in the morphology is caused by the phase separation of the polymers. Figure 3 shows the micrographs of the surface and the cross-section of the membranes containing 70/30 and 40/60 (the blends #4 and #5). According to these images, the difference between a homogeneous surface for the blend #4 membrane and pronounced globules on the surface for the blend #5 membrane is visible. Similar changes are observed in the cross-section of the membranes, where the layered structure of blend #4 are replaced by a cellular structure for blend #5. The data obtained suggest a pronounced structural change in the polymer with an increase in the proportion of PVTMS in the blend. The micrographs of other membranes are given in Appendix A (Table A1).

**Figure 4:**
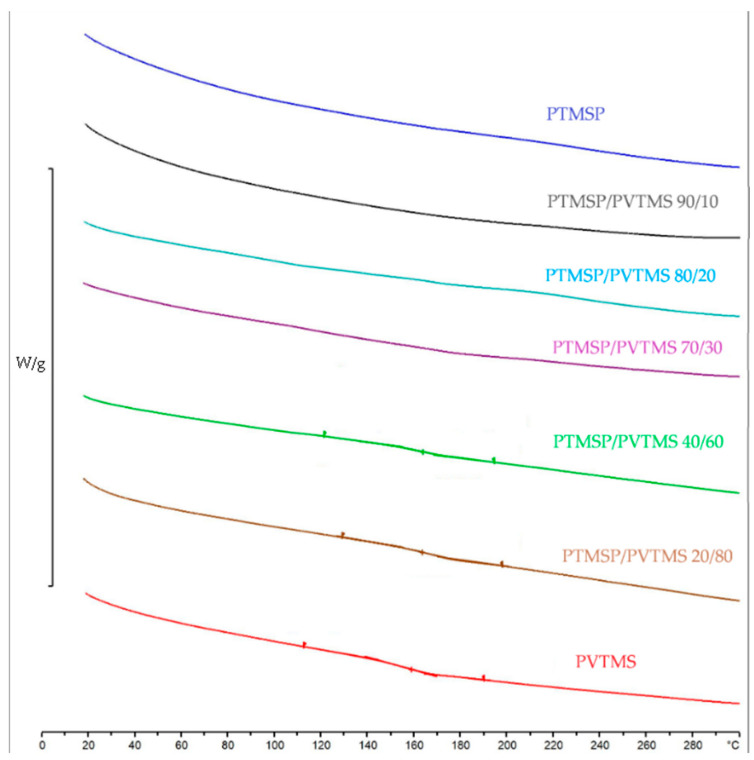
Thermograms of pure PTMSP, PVTMS samples, and PTMSP/PVTMS blends, investigated by the DSC method.

Based on the results of the DSC, a set of curves presented in Figure 4 was obtained. For PVTMS in the region of 150 °C, there is a devitrification, which is in a good agreement with the literature data (the glass transition temperature of PVTMS is 150 °C). At the same time, a transition is also observed for the blends #5 and #6, which may also indicate a different nature of the polymer. For the blends #1–4, there are no noticeable phase transitions up to 280 °C. This means that under experimental conditions (up to 100 °C), PTMSP samples with a PVTMS content of 0–30% are in a glassy state.

### 3.2. Gas Permeability

**Figure 5:**
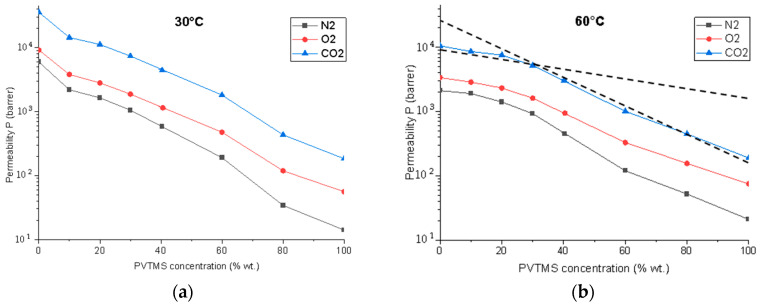
Gas permeability coefficients of membranes from PTMSP/PVTMS blends for gases O_2_, CO_2_, and N_2_ at temperatures of (**a**) 30 and (**b**) 60 °C.

The data on single gases (CO_2_, N_2_, and O_2_) permeability through dense polymer blend films, measured at 30 °C and 60 °C, are presented in Figure 5. As the PVTMS content increases, the permeability of the membranes decreases for all the gases studied. It is important to note that the dependence of the permeability coefficient in the logarithmic scale is close to linear for 30 °C, as in the case of thermodynamically compatible polymers [43]. At the same time, for 60 °C, there is a division of this dependence into two zones: from 0% to 30% PVTMS (the blends #1–4) and from 30% to 100% (the blends #5–7). In each of the zones, the dependence of the permeability coefficient in the logarithmic scale has a linear form.

The criterion of the thermodynamic compatibility of the polymers can be the value of the solubility parameter (3)

(3) $$\delta ={\left(\frac{E_{\mathrm{coh}}}{V}\right)}^{\frac{1}{2}}$$

where *E_coh_* is the cohesion energy and *V* is the molar volume of the polymer link. Malakhov et al. [37] estimated the values of the solubility parameters of PTMSP and PVTMS, which turned out to be equal to 14.5 and 14.9 MPa^1/2^ for PTMSP and PVTMS, respectively. Indeed, such close values of solubility parameters indicate the thermodynamic compatibility of these polymers.

The observed deviations of the gas permeability coefficients from the linear dependence in the logarithmic scale correlate with the SEM results and indicate the phase separation of the polymers. These differences may be associated with the polydispersity of polymer samples, that is, with a non-monomodal distribution of the polymer molecular weights.

The permeability coefficients of O_2_, CO_2_, and N_2_ for PTMSP are 9000, 35,700, and 6000 barrer, respectively. This is approximately 160, 195, and 430 times higher than the permeability values for PVTMS, respectively. Thus, the PTMSP and PVTMS blends are a convenient material for creating membranes with a wide range of gas permeability variations. In turn, the change in the dependence of the gas permeability coefficient on the composition of the PTMSP/PVTMS blend demonstrates a higher permeability for the blends up to 30% and a sharper drop after (see Figure 5 intersecting lines).

It is known that for such highly permeable glassy polymers such as PTMSP, the “physical aging” effect is especially pronounced. At the same time, there is a significant decrease in the gas permeability over time [44]. An increase in the temperature facilitates the relaxation phenomena in a nonequilibrium matrix of a glassy polymer. As a result, a higher temperature accelerates a physical aging. As can be seen from Figure 5, an increase in the temperature to 60 °C, corresponding to the maximum temperature of carbonized solvent at the outlet of the absorption column in the CO_2_ capture system, has a pronounced effect on the *p* values. As a result of the strong physical aging effect for PTMSP, its gas permeability coefficients at 60 °C are noticeably lower than at 30 °C. For example, the O_2_ permeability coefficient decreases 2.6 times from 9000 to 3400 barrer. The temperature has the opposite effect for PVTMS; an increase in the temperature from 30 °C to 60 °C leads to an increase in the oxygen permeability coefficient from 50 to 75 barrer. This is due to an increase in the diffusion coefficients of gases in PVTMS at higher temperatures and the absence of a strong physical aging effect. As a result, at 60 °C, an increase in the PVTMS content in the blend leads to a change in α(CO_2_/N_2_) from 5 to 9, while α(CO_2_/O_2_) decreases from 3.1 to 2.5.

Table 3 presents the gas permeability coefficients for the PTMSP/PVTMS blends obtained within the present study in comparison with the available data for the polymer blends based on highly permeable glassy polymers under similar conditions.

**Figure 6:**
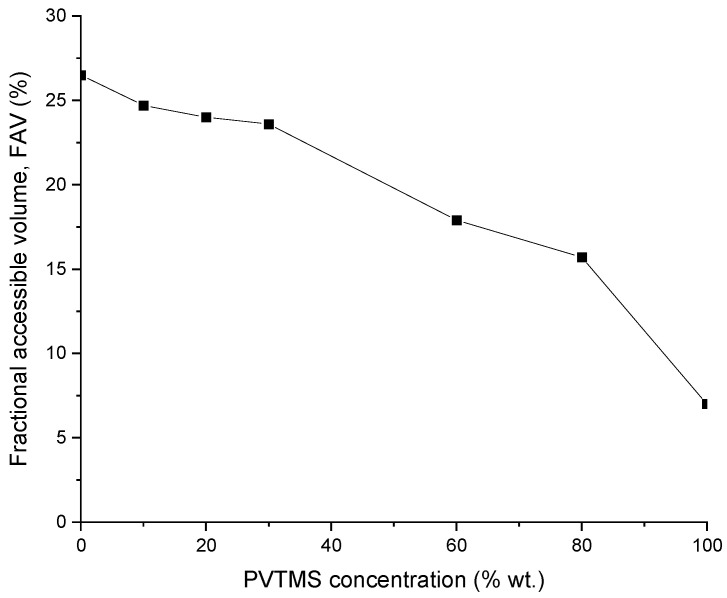
Change in the proportion of the FAV of PTMSP/PVTMS blend materials depending on the content of PVTMS.

It is known that the transport of gases in polymer membranes is determined by the fractional free volume of the polymer. In this regard, the values of fractional accessible volume (FAV) of the PTMSP/PVTMS blends were determined, as shown in Figure 6. The FAV value for pure PTMSP is 26.5%, which is in a good agreement with the literature data [41]. The addition of PVTMS leads to a monotonous decrease in the FAV value to 15.7% for the membrane # 6. It should be noted that the FAV value for a pure PVTMS is 4–7%. Thus, the decrease in the gas permeability coefficients of the studied membranes (Figure 5) is associated with a decrease in the FAV of the polymer blends in this series.

### 3.3. Gas and Water Vapor Sorption

#### 3.3.1. Gas Sorption

**Figure 7:**
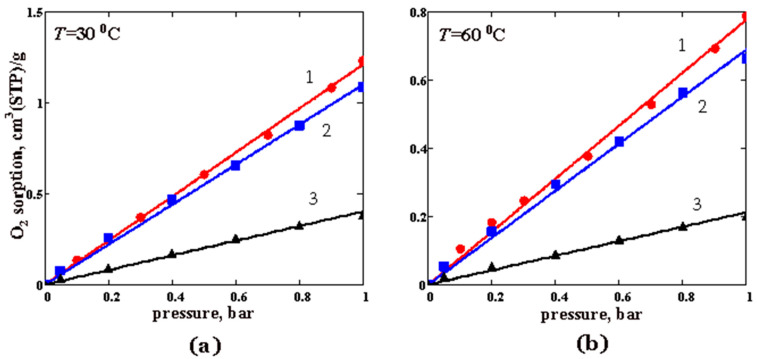
The oxygen sorption isotherms for PTMSP and PTMSP/PVTMS blends at the temperatures: (**a**) 30 °C and (**b**) 60 °C. The numbers 1, 2, and 3 denote pure PTMSP, blend #4 and blend #6, respectively. Points—experimental data, lines—approximation of the experimental data by a linear function of gas pressure.

**Figure 8:**
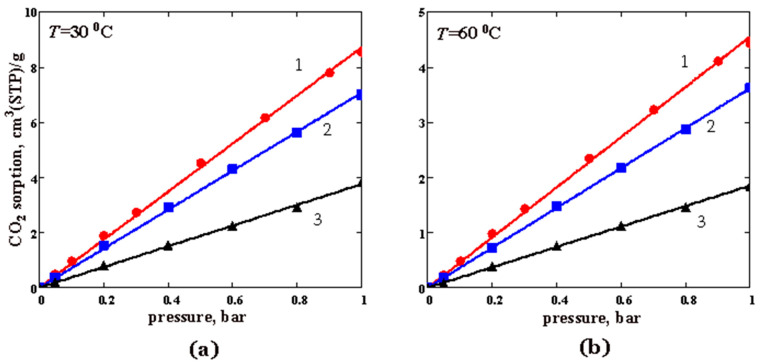
The carbon dioxide sorption isotherms for PTMSP and PTMSP/PVTMS blends at the temperatures: (**a**) 30 °C and (**b**) 60 °C. The numbers 1, 2, and 3 denote pristine PTMSP, blend #4 and blend #6, respectively. Points—experimental data, lines—approximation of the experimental data by a linear function of gas pressure.

The sorption isotherms for O_2_ and CO_2_ in pure PTMSP and PTMSP/PVTMS blends are shown in Figure 7and Figure 8, respectively. In the studied pressure range, the isotherms are linear. As is seen from Figure 7, with an increase in the PVTMS content in the blend, the oxygen sorption decreases. Blend #3 contains 80 wt% PVTMS sorbs three times less oxygen than PTMSP at 30 °C (Figure 7a). At 60 °C, the difference becomes even greater (Figure 7b). As expected, the oxygen sorption in the polymer films decreases with an increasing temperature, which corresponds to the exothermic sorption process (Figure 7). Similar conclusions apply to the CO_2_ sorption (Figure 8). The sample of pure PTMSP (sample 1) absorbs 2.3–2.5 times more CO_2_ than blend #3 with 80 wt% of the PVTMS content.

The solubility coefficients (*S*) for both gases are given in Table 4. The *S* values decrease as the fraction of PVTMS in its blends with PTMSP increases, and as the temperature rises. The decrease in the gas solubility is associated with the compaction (densification) of PTMSP when blended with PVTMS [37] and, as a result, with a decrease in the available “sorption sites” (or free volume elements) for the sorbed gas. In this work, we found that the ratio of the solubility coefficients for CO_2_ and O_2_, i.e., a so-called ideal solubility selectivity, varies non-monotonically with an increasing PVTMS content in the blend PTMSP/PVTMS. This ratio slightly decreases at a 30% PVTMS content and then increases to values of about 9 at an 80% PVTMS content in the blend (Table 4).

#### 3.3.2. Water Vapor Sorption

**Figure 9:**
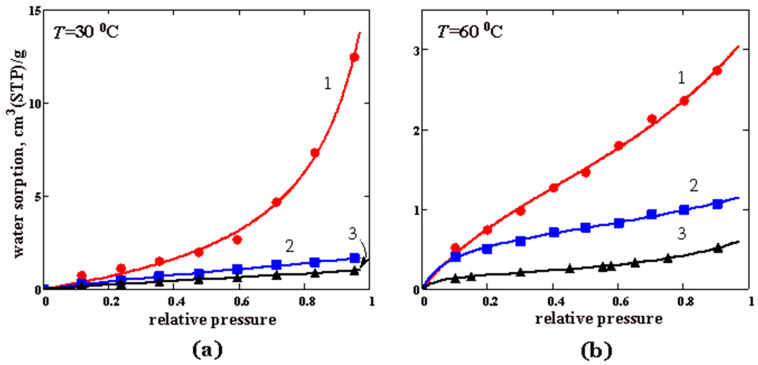
Water sorption isotherms for PTMSP and PTMSP/PVTMS blends at 30 °C (**a**) and 60 °C (**b**). The numbers 1, 2, and 3 denote pristine PTMSP, blend #4 and blend #6, respectively. Points—experimental data, lines—approximation of the experimental data by Equation (4).

The results of the measurement of the water vapor sorption isotherms in the PTMSP/PVTMS blends are shown in Figure 9. Unlike the O_2_ and CO_2_ sorption isotherms, the obtained isotherms are nonlinear and characterized by the point of inflection (this is more pronounced at 60 °C in Figure 9b). Because of the ability of the water molecules to form hydrogen bonds, one can assume that this indicates the multimolecular sorption of water in the examined glassy polymers. Note that a self-association or clustering phenomena are often observed during water vapor sorption in glassy polymers [30,46].

Water vapor sorption *V* at a given relative pressure *a* = *p*/*p*_0_ (*p*_0_ is the saturated pressure of the bulk liquid) was described by the modified BET equation [47], also called the GAB (Guggenheim–Anderson–de Boer) isotherm (see, e.g., [48]):

(4) $$V=V_{m}\frac{Ka}{\left(1-K_{as}a\right)\left(1+(K-K_{as})a\right)}$$

Here, *V_m_* is the monolayer sorption capacity, *K* is the binding constant describing the sorbate–sorbent interaction, and *K_as_* is the binding constant describing the sorbate–sorbate interaction in the side associate. The calculated parameters are listed in Table 5.

As can be seen from Figure 9a, the water sorption at 30 °C is low at *p*/*p*_0_ < 0.2 and almost independent of PVTMS content in the samples. At *p*/*p*_0_ > 0.2, the water sorption in PTMSP increases sharply, in contrast to the samples containing PVTMS, and reaches values comparable to and even exceeding the CO_2_ sorption at the same temperature (Figure 8a). An increase in the temperature to 60 °C (Figure 9b) leads to a regular decrease in the water sorption, while blend #6 demonstrates the lowest sorption. As noted above, the density of the PTMSP/PVTMS blends is larger than that of pristine PTMSP. Consequently, a smaller fraction of the free volume is available for the sorption of water molecules in the PTMSP/PVTMS blends, which explains the lower water sorption in these blends compared to pristine PTMSP.

### 3.4. Transport of Aqueous Alkanolamine Solvent Vapors

**Figure 10:**
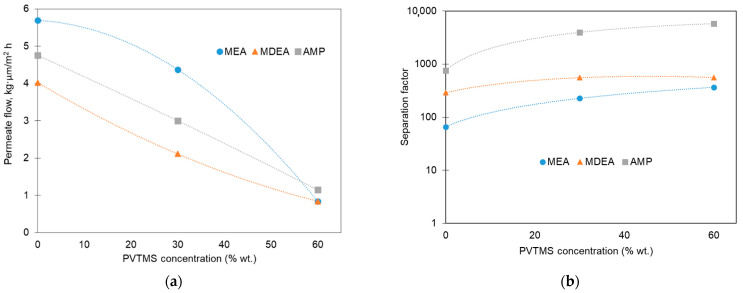
TPV results for model solvents (aqueous solutions of MEA (30% wt.), MDEA (40% wt.), and AMP (30% wt.) in distilled water) at T = 60 °C: (**a**) permeate (solvent vapors) flows, normalized by the membrane thickness, and (**b**) separation factors.

Since the membrane contactor which should be applied to remove dissolved O_2_ from the rich amine solution has a typical temperature no more than 60 °C, the fabricated polymer blend membranes were tested for the vapor permeation of aqueous alkanolamine solutions at *T* = 60 °C. Figure 10 shows permeate flows (Figure 10a) and the amine/water separation factor (Figure 10b) as a function of the PVTMS content in the polymer blend. It can be seen that the solvent loss due to its evaporation and permeation through the membrane can be decreased by a factor of 4–6 by introducing up to 60% of PVTMS. With the respect to the type of amine used, the normalized permeate flow values changed in the following order: MEA (0.8–5.7 kg⋅µm⋅m^−2^⋅h^−1^) > AMP (1.1–4.8 kg⋅µm⋅m^−2^⋅h^−1^) > MDEA (0.8–4.0 kg⋅µm⋅m^−2^⋅h^−1^). These results can be explained by the corresponding drop in the saturated vapor pressures of the studied amines (see for example [6,49]), and an increase in their molecular weight (kinetic diameter). It should be noticed that the presence of PVTMS in the membrane matrix resulted in a more pronounced reduction in the permeation of amine molecules compared with water ones. For instance, by the replacement of PTMSP with blend #5, it was possible to increase the water/amine separation factor from 66 to 370, from 290 to 560, and from 750 to 5790 for the MEA, MDEA, and AMP solvents, respectively. The increase in the separation factor indicates the significant reduction in the loss of alkanolamine due to the evaporation during the deoxygenation process.

It is noteworthy that the separation factor increases most strongly during the transition from the PTMSP to blend #4 (Figure 10b). Its values for blend #4 are 226, 555, and 3968 for the aqueous solutions of MEA, MDEA, and AMP, respectively. Thus, when 30% PVTMS is introduced into PTMSP, it leads to an increase in the separation factor by 3.4, 1.9, and 5.3 times for MEA, MDEA, and AMP, respectively. At the same time, a further increase in the PVTMS content from 30 to 60% increases the separation factor, for example, MEA only 1.6 times from 226 to 290. Considering the sharp drop in the oxygen permeability coefficient by 9 times from 900 to 100 barrer during the transition from blend #4 to blend #5 (Figure 5b), blend #4 appears to be the preferred material of the composite membrane designed for the O_2_ removal from the amine solvents. This composition provides an optimal combination of sufficiently high oxygen permeability values and the amine/water separation factor at 60 °C (the maximum temperature of carbonized solvent at the outlet of the absorption column). Thus, it can be expected that a composite membrane with a selective layer from blend #4 will allow for an effective deoxygenation of the solvent in a membrane contactor with insignificant losses of alkanolamine.

## 4. Conclusions

The dense membranes made of blends of the glassy polymers PTMSP and PVTMS perspective as selective layers for the deoxygenation of alkanolamine CO_2_ solvents in gas–liquid composite membrane contactors have been studied. The permeability of O_2_, CO_2,_ and N_2_ through dense films of the PTMSP/PVTMS blends with the PVTMS content was studied at 0, 10, 20, 30, 40, 60, 80, and 100% at temperatures of 30 and 60 °C. As the PVTMS content in the blend increases, the O_2_ and CO_2_ permeability coefficients at 30 °C decrease by 160 and 195 times, respectively. It is shown that the form of the dependences of the permeability coefficients of the studied gases on the PVTMS content in the PTMSP/PVTMS blend is close to linear in the logarithmic scale in the range from 0% to 30% and in the range from 30% to 100%, however, there are differences in the form of dependence for these ranges. In particular, a sharper decrease in the permeability coefficient was observed for the zone after 30%. These results are also consistent with the visual representation in the microphotographs. The results on the gas permeability are consistent with the values of the fractional free volume of the polymer blends. Thus, the addition of PVTMS leads to a monotone decrease in the FAV value from 26.5% for pure PTMSP to 15.7% for a blend of PTMSP/PVTMS of a composition of 20/80. The PTMSP/PVTMS blends are a convenient material for creating membranes with controlled characteristics in a wide range of gas permeability variations.

As a result of the strong physical aging effect for PTMSP, its gas permeability coefficients at 60 °C are noticeably lower than at 30 °C. The coefficient of the O_2_ permeability decreases by 2.6 times from 9000 to 3400 barrer. At the same time, an increase in the temperature leads to an increase in the gas permeability coefficients for PVTMS. As a result, at 60 °C, an increase in the PVTMS content in the blend leads to a decrease in the ideal selectivity of α(CO_2_/O_2_) from 3.1 to 2.5. This should have a positive effect on the competitive transfer of CO_2_ compared to O_2_.

The sorption isotherms of O_2_, CO_2,_ and water vapor were measured in pure PTMSP and the PTMSP/PVTMS blends 70/30 and 20/80 at 30 and 60 °C. Unlike the water vapor sorption, gas sorption isotherms are linear in nature. An increase in the temperature from 30 to 60 °C leads to a decrease in the gas solubility coefficients, which is consistent with the data on gas permeability. The isotherms of the water vapor sorption are nonlinear in nature and can be described by the modified BET equation.

The transport properties of membranes based on the PTMSP/PVTMS blends toward solvent vapors were determined in the thermo-pervaporation mode using aqueous solutions of MEA (30%), MDEA (40%), and AMP (30%) at a temperature of 60 °C. The membranes demonstrated high pervaporation separation factors for water, which suggests negligible amine losses during the deoxygenation of solvents in the membrane contactor. A joint analysis of the TPV data and gas permeabilities of the polymer blends allowed us to conclude that the composition of PTMSP/PVTMS 70/30 provides an optimal combination of sufficiently high values of oxygen permeability and the pervaporation separation factor at 60 °C. Thus, it can be expected that the PTMSP/PVTMS 70/30 blend is a promising material for the development of new composite membranes designed for the deoxygenation of CO_2_ capture amine solvents in gas–liquid membrane contactors.

## Figures and Tables

**Table 1:** Composition and kinetic diameters of gas molecules that make up a typical flue gas of a coal-fired power plant [7].

The Molecule	Concentration, vol.%	Kinetic Diameter, Å
CO_2_	15–16	3.30
N_2_	70–75	3.64
H_2_O	5–7	2.65
O_2_	3–4	3.45
CO	<1	3.75

**Table 2:** Composition and thickness of the studied polymer blend membranes.

Blend #	The Ratio of PTMSP/PVTMS Fractions in the Membrane, %	Film Thickness, Microns
1	100/0	36
2	90/10	30
3	80/20	30
4	70/30	30
5	40/60	31
6	20/80	33
7	0/100	25

**Table 3:** The gas permeability and selectivity data for highly permeable glassy polymer blends.

Polymer Blend	Source	T/P (°C/atm)	P_N2_, Barrer	P_CO2_, Barrer	α(CO_2_/N_2_)
PTMSP/PVTMS 70/30	This work	30/0.2–0.8	1050	7324	6.9
	[37]	30/0.2–0.8	990	5772	5.8
PIM-1/Ultem 70/30	[36]	30/0.2–0.8	21.9	477.1	21.8
cPIM-1/Matrimid 70/30	[45]	35/3.5	21	486	23.1

**Table 4:** Solubility coefficients (Henry’s constants) of oxygen and carbon dioxide in PTMSP and PTMSP/PVTMS blends (cm^3^ (STP)/g·bar), and CO_2_/O_2_ ideal solubility selectivity.

Polymer	S(O_2_)		S(CO_2_)		S(CO_2_)/S(O_2_)	
	30 °C	60 °C	30 °C	60 °C	30 °C	60 °C
PTMSP	1.210	0.777	8.696	4.543	7.2	5.8
Blend #4	1.099	0.689	7.057	3.611	6.4	5.2
Blend #6	0.402	0.212	3.747	1.847	9.3	8.7

**Table 5:** Water sorption parameters of Equation (1) for PTMSP and PTMSP/PVTMS blends at 30 °C and 60 °C.

Polymer	V_m_ (cm^3^(STP)/g)		K		K_as_	
	30 °C	60 °C	30 °C	60 °C	30 °C	60 °C
PTMSP	2.648	1.503	1.093	3.516	0.86	0.58
Blend #4	1.509	0.665	1.533	11.744	0.38	0.46
Blend #6	1.036	0.180	1.269	21.902	0.34	0.725

## Data Availability

The data presented in this study are available on request from the corresponding author.

## Appendix A

**Table A1:** Micrographs of the surface and cross-section of the studied membranes.

The Ratio of PTMSP/PVTMS Fractions in the Membrane, %	SEM Images of Membrane Surfaces	SEM Images of Membrane Cross-Section
100/0		
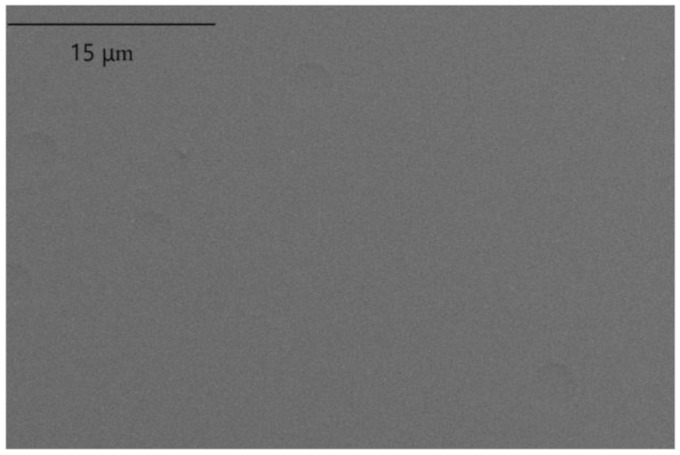
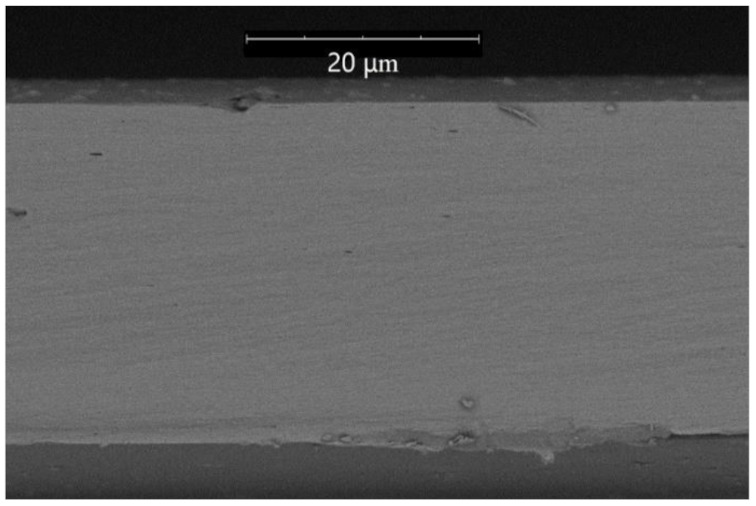
90/10		
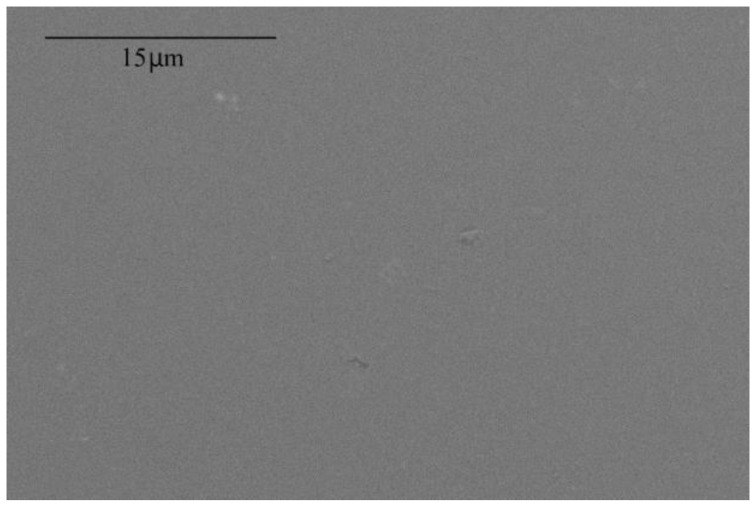
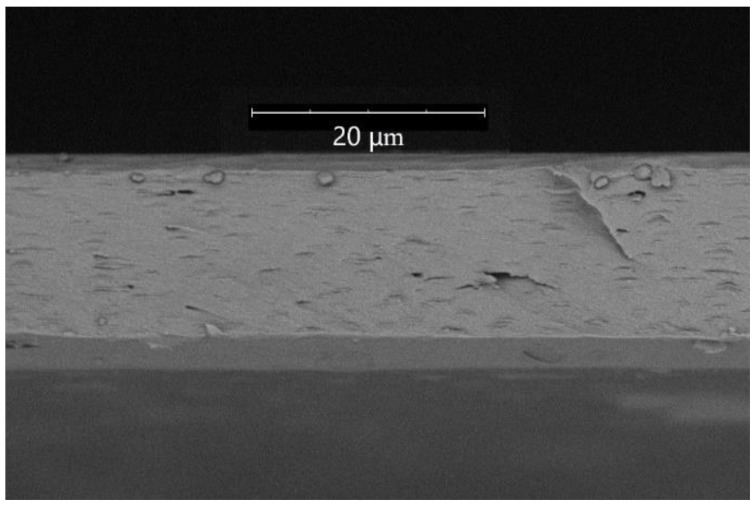
80/20		
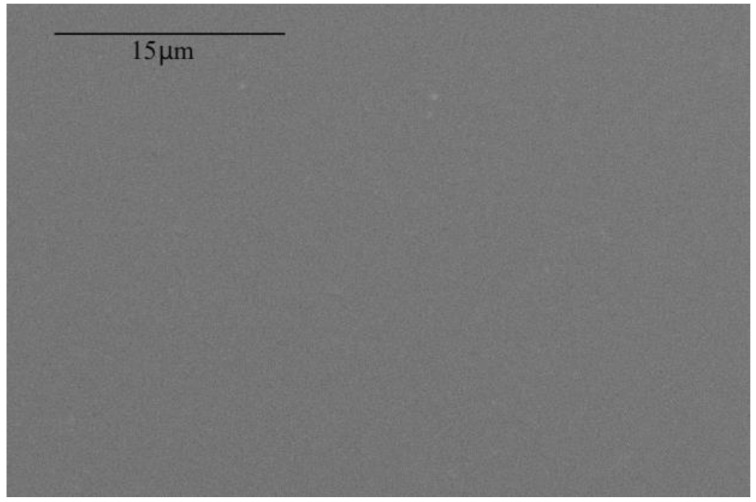
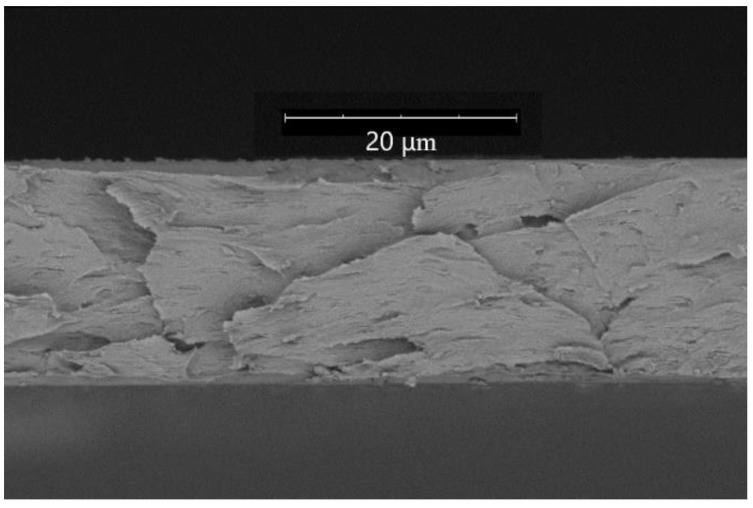
70/30		
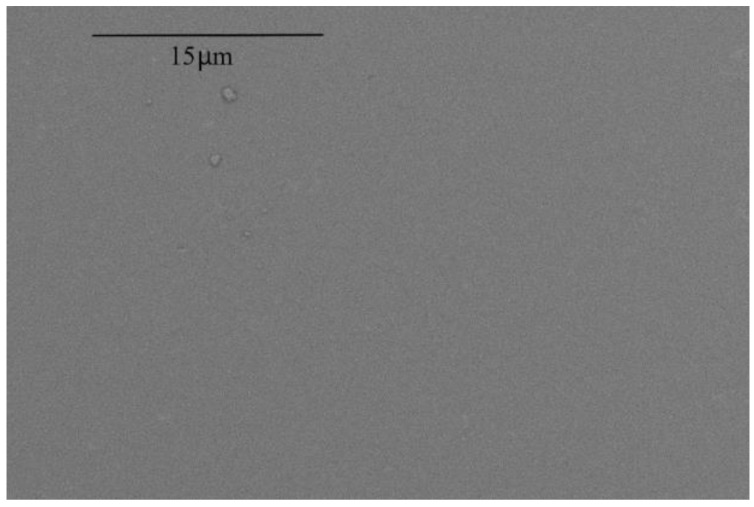
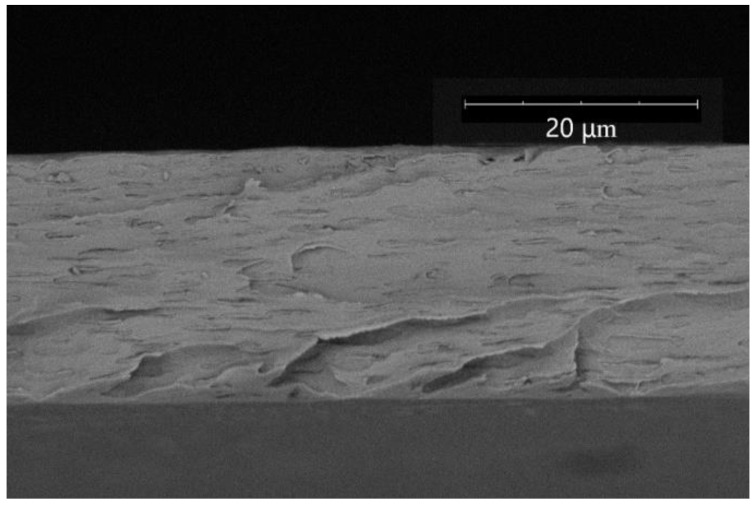
40/60		
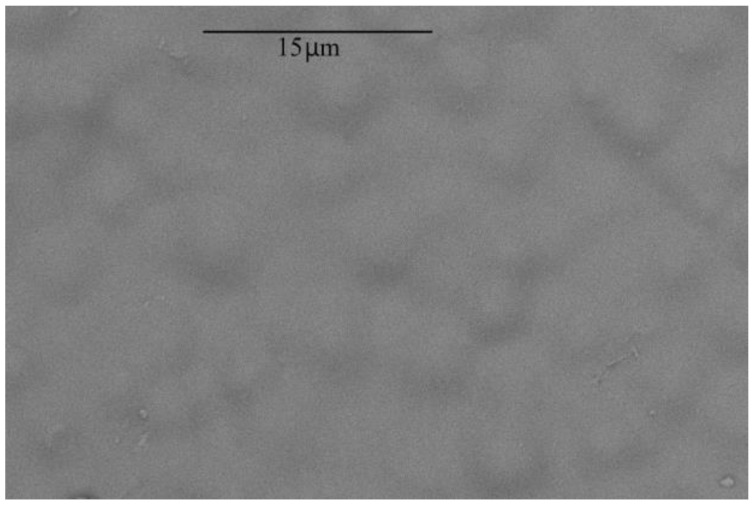
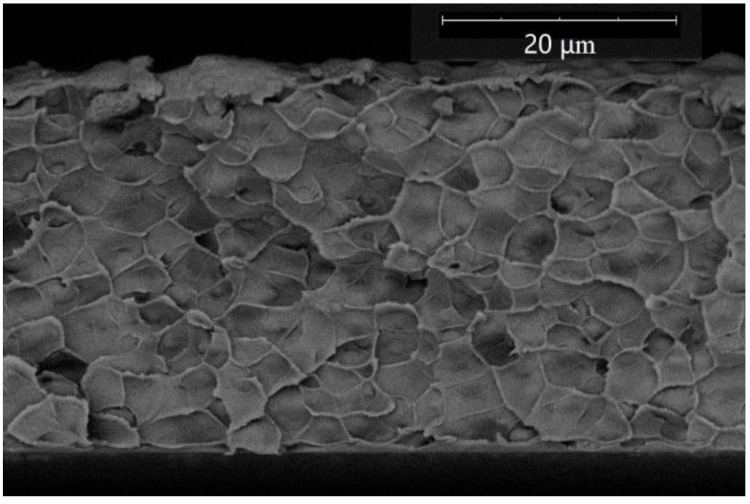
20/80		
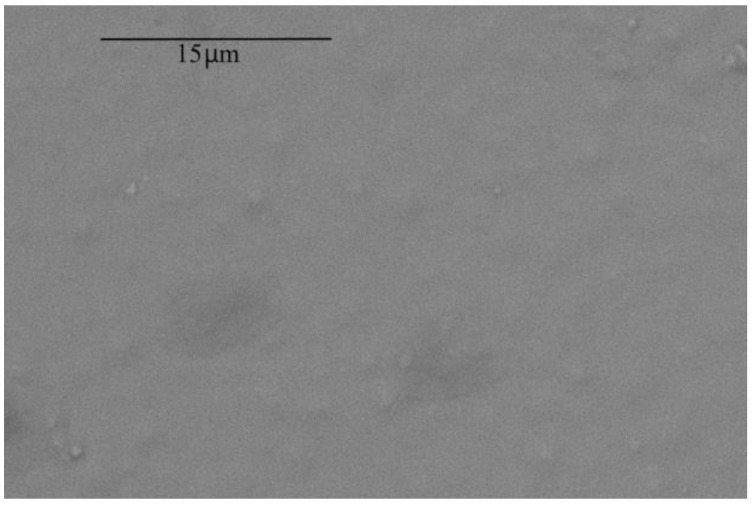
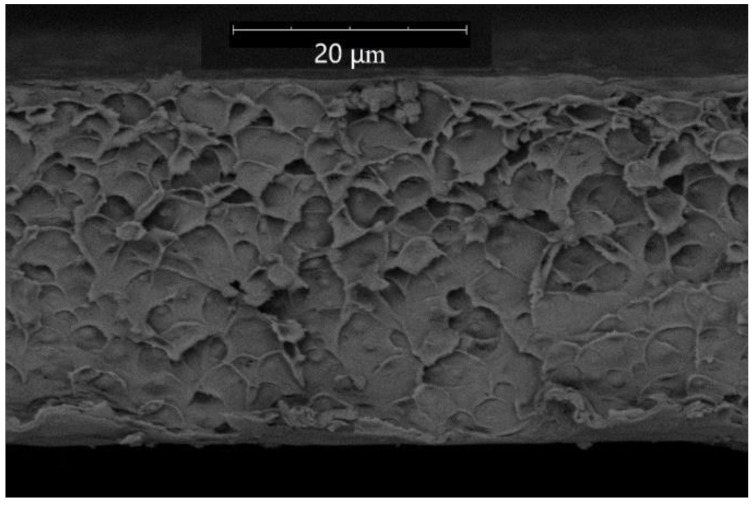
0/100		

## References

[1] Hansen J., Johnson D., Lacis A., Lebedeff S., Lee P., Rind D., Russell G. (1981). Climate Impact of Increasing Atmospheric Carbon Dioxide. Science.

[2] 2011 Leaders’ Declaration. https://www.apec.org/meeting-papers/leaders-declarations/2011/2011_aelm.

[3] Bazhenov S., Chuboksarov V., Maximov A., Zhdaneev O. (2022). Technical and Economic Prospects of CCUS Projects in Russia. Sustain. Mater. Technol..

[4] Bazhenov S.D., Novitskii E.G., Vasilevskii V.P., Grushevenko E.A., Bienko A.A., Volkov A.V. (2019). Heat-Stable Salts and Methods for Their Removal from Alkanolamine Carbon Dioxide Absorbents. Russ. J. Appl. Chem..

[5] Buvik V., Høisæter K.K., Vevelstad S.J., Knuutila H.K. (2021). A Review of Degradation and Emissions in Post-Combustion CO_2_ Capture Pilot Plants. Int. J. Greenh. Gas Control.

[6] Kohl A.L., Nielsen R.B. (1997). Gas Purification 5th Ed. Houst. Gulf Publ. Co..

[7] Chen Z., Deng S., Wei H., Wang B., Huang J., Yu G. (2013). Activated Carbons and Amine-Modified Materials for Carbon Dioxide Capture—A Review. Front. Environ. Sci. Eng..

[8] Popoola L.T., Grema A.S., Latinwo G.K., Gutti B., Balogun A.S. (2013). Corrosion Problems during Oil and Gas Production and Its Mitigation. Int. J. Ind. Chem..

[9] Xiang Y., Xie W., Ni S., He X. (2020). Comparative Study of A106 Steel Corrosion in Fresh and Dirty MEA Solutions during the CO_2_ Capture Process: Effect of NO_3_^−^. Corros. Sci..

[10] Choi Y.-S., Duan D., Nesic S., Vitse F., Bedell S.A., Worley C. (2010). Effect of Oxygen and Heat Stable Salts on the Corrosion of Carbon Steel in MDEA-Based CO_2_ Capture Process. Corrosion.

[11] Zheng L., Landon J., Matin N.S., Thomas G.A., Liu K. (2016). Corrosion Mitigation via a PH Stabilization Method in Monoethanolamine-Based Solutions for Post-Combustion CO_2_ Capture. Corros. Sci..

[12] Soosaiprakasam I.R., Veawab A. (2008). Corrosion and Polarization Behavior of Carbon Steel in MEA-Based CO_2_ Capture Process. Int. J. Greenh. Gas Control.

[13] Kladkaew N., Idem R., Tontiwachwuthikul P., Saiwan C. (2011). Studies on Corrosion and Corrosion Inhibitors for Amine Based Solvents for CO_2_ Absorption from Power Plant Flue Gases Containing CO_2_, O_2_ and SO_2_. Energy Procedia.

[14] Moser P., Wiechers G., Schmidt S., Monteiro J.G.M.-S., Charalambous C., Garcia S., Fernandez E.S. (2020). Results of the 18-Month Test with MEA at the Post-Combustion Capture Pilot Plant at Niederaussem–New Impetus to Solvent Management, Emissions and Dynamic Behaviour. Int. J. Greenh. Gas Control.

[15] Gouedard C., Picq D., Launay F., Carrette P.-L. (2012). Amine Degradation in CO_2_ Capture. I. A Review. Int. J. Greenh. Gas Control.

[16] Wang T., Hovland J., Jens K.J. (2015). Amine Reclaiming Technologies in Post-Combustion Carbon Dioxide Capture. J. Environ. Sci..

[17] Goff G.S., Rochelle G.T. (2006). Oxidation Inhibitors for Copper and Iron Catalyzed Degradation of Monoethanolamine in CO_2_ Capture Processes. Ind. Eng. Chem. Res..

[18] Sexton A.J., Rochelle G.T. (2009). Catalysts and Inhibitors for Oxidative Degradation of Monoethanolamine. Int. J. Greenh. Gas Control.

[19] Alent’ev A.Y., Volkov A.V., Vorotyntsev I.V., Maksimov A.L., Yaroslavtsev A.B. (2021). Membrane Technologies for Decarbonization. Membr. Membr. Technol..

[20] Bazhenov S.D. (2022). Prospects for Membrane Deoxygenation of Alkanolamine CO_2_ Solvents to Prevent Their Degradation (A Minireview). Pet. Chem..

[21] Sengupta A., Peterson P.A., Miller B.D., Schneider J., Fulk C.W. (1998). Large-Scale Application of Membrane Contactors for Gas Transfer from or to Ultrapure Water. Sep. Purif. Technol..

[22] Peng Z.-G., Lee S.-H., Zhou T., Shieh J.-J., Chung T.-S. (2008). A Study on Pilot-Scale Degassing by Polypropylene (PP) Hollow Fiber Membrane Contactors. Desalination.

[23] Kattan O., Ebbers K., Koolaard A., Vos H., Bargeman G. (2018). Membrane Contactors: An Alternative for de-Aeration of Salt Solutions?. Sep. Purif. Technol..

[24] Martić I., Maslarević A., Mladenović S., Lukić U., Budimir S. (2015). Water Deoxygenation Using Hollow Fiber Membrane Module with Nitrogen as Inert Gas. Desalination Water Treat..

[25] Bhaumik D., Majumdar S., Fan Q., Sirkar K.K. (2004). Hollow Fiber Membrane Degassing in Ultrapure Water and Microbiocontamination. J. Membr. Sci..

[26] Li T., Yu P., Luo Y. (2015). Deoxygenation Performance of Polydimethylsiloxane Mixed-matrix Membranes for Dissolved Oxygen Removal from Water. J. Appl. Polym. Sci..

[27] Malakhov A.O., Bazhenov S.D., Vasilevsky V.P., Borisov I.L., Ovcharova A.A., Bildyukevich A.V., Volkov V.V., Giorno L., Volkov A.V. (2019). Thin-Film Composite Hollow Fiber Membranes for Ethylene/Ethane Separation in Gas-Liquid Membrane Contactor. Sep. Purif. Technol..

[28] Dibrov G.A., Volkov V.V., Vasilevsky V.P., Shutova A.A., Bazhenov S.D., Khotimsky V.S., Van de Runstraat A., Goetheer E.L.V., Volkov A.V. (2014). Robust High-Permeance PTMSP Composite Membranes for CO_2_ Membrane Gas Desorption at Elevated Temperatures and Pressures. J. Membr. Sci..

[29] Trusov A., Legkov S., van den Broeke L.J., Goetheer E., Khotimsky V., Volkov A. (2011). Gas/Liquid Membrane Contactors Based on Disubstituted Polyacetylene for CO_2_ Absorption Liquid Regeneration at High Pressure and Temperature. J. Membr. Sci..

[30] Scholes C.A., Jin J., Stevens G.W., Kentish S.E. (2015). Competitive Permeation of Gas and Water Vapour in High Free Volume Polymeric Membranes. J. Polym. Sci. Part B Polym. Phys..

[31] Hu Y., Shiotsuki M., Sanda F., Freeman B.D., Masuda T. (2008). Synthesis and Properties of Indan-Based Polyacetylenes That Feature the Highest Gas Permeability among All the Existing Polymers. Macromolecules.

[32] Mushtaq A., Mukhtar H.B., Shariff A.M., Mannan H.A. (2013). A Review: Development of Polymeric Blend Membrane for Removal of CO_2_ from Natural Gas. Int. J. Eng..

[33] Yong W.F., Zhang H. (2021). Recent Advances in Polymer Blend Membranes for Gas Separation and Pervaporation. Prog. Mater. Sci..

[34] Mannan H.A., Mukhtar H., Murugesan T., Nasir R., Mohshim D.F., Mushtaq A. (2013). Recent Applications of Polymer Blends in Gas Separation Membranes. Chem. Eng. Technol..

[35] Robeson L.M. (2010). Polymer Blends in Membrane Transport Processes. Ind. Eng. Chem. Res..

[36] Hao L., Li P., Chung T.-S. (2014). PIM-1 as an Organic Filler to Enhance the Gas Separation Performance of Ultem Polyetherimide. J. Membr. Sci..

[37] Malakhov A.O., Dibrov G.A., Litvinova E.G., Novitsky E.G. (2015). Gas Permeability of Homogeneous and Composite Membranes Based on Poly (Trimethylsilylpropyne)/Poly (Vinyltrimethylsilane) Blends. Pet. Chem..

[38] Malakhov A.O., Bazhenov S.D. (2018). Carbon Dioxide Desorption from Amine Solution in a Nonporous Membrane Contactor. Pet. Chem..

[39] Platé N., Yampol’skii Y. (2018). Relationship between structure and transport properties for high free volume polymeric materials. Polymeric Gas Separation Membranes.

[40] Volkov A.V., Fedorov E.V., Malakhov A.O., Volkov V.V. (2002). Vapor Sorption and Dilation of Poly [(1-Trimethylsilyl)-1-Propyne] in Methanol, Ethanol, and Propanol. Polym. Sci. Ser. BCC Vysokomol. Soedin..

[41] Yushkin A., Grekhov A., Matson S., Bermeshev M., Khotimsky V., Finkelstein E., Budd P.M., Volkov V., Vlugt T.J., Volkov A. (2015). Study of Glassy Polymers Fractional Accessible Volume (FAV) by Extended Method of Hydrostatic Weighing: Effect of Porous Structure on Liquid Transport. React. Funct. Polym..

[42] Myers A.L., Monson P.A. (2014). Physical Adsorption of Gases: The Case for Absolute Adsorption as the Basis for Thermodynamic Analysis. Adsorption.

[43] Nagai K., Kanehashi S., Tabei S., Nakagawa T. (2005). Nitrogen Permeability and Carbon Dioxide Solubility in Poly (1-Trimethylsilyl-1-Propyne)-Based Binary Substituted Polyacetylene Blends. J. Membr. Sci..

[44] Lau C.H., Nguyen P.T., Hill M.R., Thornton A.W., Konstas K., Doherty C.M., Mulder R.J., Bourgeois L., Liu A.C., Sprouster D.J. (2014). Ending Aging in Super Glassy Polymer Membranes. Angew. Chem. Int. Ed..

[45] Yong W.F., Chung T.-S. (2015). Miscible Blends of Carboxylated Polymers of Intrinsic Microporosity (CPIM-1) and Matrimid. Polymer.

[46] Arce A., Fornasiero F., Rodríguez O., Radke C.J., Prausnitz J.M. (2004). Sorption and Transport of Water Vapor in Thin Polymer Films at 35 C. Phys. Chem. Chem. Phys..

[47] Brunauer S., Skalny J., Bodor E.E. (1969). Adsorption on Nonporous Solids. J. Colloid Interface Sci..

[48] Vopička O., Randová A., Friess K. (2014). Sorption of Vapours and Liquids in PDMS: Novel Data and Analysis with the GAB Model of Multilayer Adsorption. Eur. Polym. J..

[49] Klepácová K., Huttenhuis P.J., Derks P.W., Versteeg G.F. (2011). Vapor Pressures of Several Commercially Used Alkanolamines. J. Chem. Eng. Data.

